# Supplementary material for: Chromothripsis during telomere crisis is independent of NHEJ, and consistent with a replicative origin
Source: Genome Res. 2019 May;29(5):737–49. doi: 10.1101/gr.240705.118 (PMC6499312; doi:10.1101/gr.240705.118)
Supplement: Supplemental Material [file supp_gr.240705.118_Supplemental_file_1.zip › contigs/annotated_contigs/DB73/contig.2.DB73_length_436_mean_cov_6.20183486239.docx]

**DB73_length_436_mean_cov_6.20183486239**

```
TTTCTTTCTTTCTTTCTTTCTTTCTTTCTTTCTTTCTTTCTTTCTCTTTCCTTTCTTTCTTTCTTTCTTTCTTTC|TTTCTTTCTTTCT
 >chr19:58938666-58938773 + E=7e-53 p=0e+00                                 >chr6:113730
TTCTTTCTTTCTTTCTTTC|TTTCTTCTTTCTTTTTTGACACAGTCTCCCTATGTCACCCAGGCTGGAGTGCAGTGGTGGAATTATGGC
324-113730685 - E=4e-203
AATCTTGAACTCTTGCCTCAGCCTCCCTAGTAGCTGGGACTACAGGCATGCACCACCATGCCTAGCTACTGTTTCTGTTGGATTGTTTT

TGTTTTTGTAGAGGTGGGGTCTCATTATGTTGCCCAAGCTGGACTTGAAGTCCTTGGCTCCAGTGATCTTCCTTCCTCAGGCTCCTGAA

ATGCTGGGAGTATAGGTGTGAGCCACCATACCCAGCCCTGTACAATTTTTAGATTTGAATTTGTAAAGTGTGTCTTGATGGA
```
